# Searching for vertically transmitted endosymbionts in over 4,000 wild strains of three self-fertilizing *Caenorhabditis* species

**DOI:** 10.17912/micropub.biology.002151

**Published:** 2026-06-08

**Authors:** Lance M. O'Connor, Anupama Singh, Robert J. Luallen, Gaotian Zhang, Erik C. Andersen

**Affiliations:** 1 Department of Biology, Johns Hopkins University, Baltimore, MD, US; 2 Cell, Molecular, Developmental Biology, and Biophysics doctoral program, Johns Hopkins University, Baltimore, MD, US; 3 Department of Biology, San Diego State University, San Diego, CA, US; 4 Institut de Biologie de l’École Normale Supérieure, CNRS, INSERM, Paris, France

## Abstract

Vertically transmitted endosymbionts are microbes that live inside host cells and are transmitted between parent and offspring. Free-living nematodes interact with a wide variety of microbes but no vertically transmitted microbes have been discovered. To identify vertically transmitted microbes in *Caenorhabditis briggsae* , *Caenorhabditis elegans* , and *Caenorhabditis tropicalis* wild strains, we isolated DNA and sequenced over 4,000 different wild strains where horizontally transmitted microbes were removed. Then, we taxonomically classified unaligned sequence reads and experimentally probed strains with possible vertically transmitted microbes. We found no experimental evidence of vertically transmitted microbes in any of these strains.

**Figure 1. Detection of potential vertically transmitted microbes in self-fertilizing Caenorhabditis wild strains:** **A.** Heatmap displaying the detection of *Nematocida* in microsporidia-infected strains (control, first two panels) and selected genera across self-fertilizing *Caenorhabditis* species. 10% of minimizers (non-redundant k-mers) created from unmapped reads for a given strain must match genome sequences from a genus to be classified as detected. Nine strains with detectable levels of possible vertically transmitted microbe sequence reads are displayed for each self-fertilizing species. **B.** Representative FISH images of wild *C. elegans* or *C. briggsae* strains probed with EUB338, which is a universal probe that recognizes a conserved loop in V1 of the 16S rRNA in a diverse array of bacterial taxa. As a positive control for the FISH procedure, we used N2 *C. elegans* grown on MYb71 ( *Ochrobactrum pecoris* ) and punctate bacterial staining can be observed. Scale bars are 20 µm.

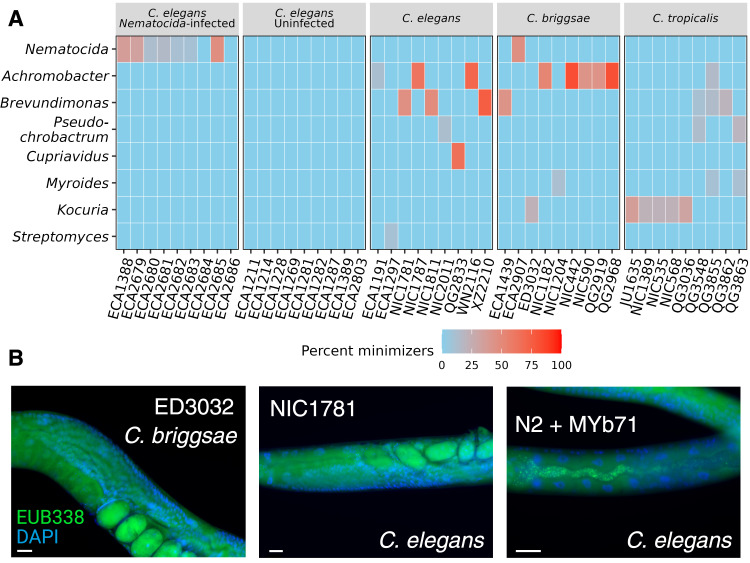



## Description

Extensive sampling of wild *Caenorhabditis* and other free-living nematodes has uncovered a vast array of microbes that commonly interact with them, including extracellular bacteria and fungi, single-strand positive-sense RNA viruses, and eukaryotic parasites (Dirksen et al., 2016; Osman et al., 2018; Troemel et al., 2008). Recently, the first intracellular bacteria were discovered in free-living nematodes by phenotypic characterization of intracellular intestinal infections using light microscopy and confirmed using fluorescence *in situ* hybridization (FISH) microscopy and transmission electron microscopy (Tran et al., 2022). The identification of colonization or infection followed by techniques for molecular validation has been commonly used to find new microbial infections in wild-caught nematodes. However, vertically transmitted endosymbionts have been notably absent in the microbiota of free-living nematodes.

Vertically transmitted endosymbionts are microbes that live inside host cells and are transmitted between parent and offspring. In animals, these microbes are often unable to survive outside of the host and are transmitted by the colonization of the germline. *Wolbachia pipientis* is likely the best studied vertically transmitted bacteria, which infects most insect species and has been designed for mosquito control (Hague et al., 2024). *Wolbachia* is found in many filarial nematode species and is considered essential for normal development and fertility (Taylor et al., 2005). However, we have yet to find vertically transmitted microbes in free-living nematodes, possibly because the phenotype-based discovery pipeline described above might overlook symbionts that do not generate obvious infection signatures observed using microscopy.

Over the last 20 years, advances in high-throughput DNA sequencing have been enabled by improvements to every step of the process from DNA extraction and preparation to base-calling accuracy. Nonetheless, sample contamination by DNA from unknown organisms sequenced along with the targeted sample of interest remains a persistent challenge. When preparing *Caenorhabditis* strains for sequencing, plates containing adult worms are cleaned using bleach, dissolving their hypodermis and releasing embryos. These embryos are grown into large mixed-stage populations that are collected, DNA isolated, and whole-genome sequenced (Crombie et al., 2024). This process will theoretically remove all non-vertically transmitted microbial contamination. The nematode community has collected thousands of *Caenorhabditis* strains from all over the world (Crombie et al., 2024; Lee et al., 2021; Moya et al., 2025). The genomes of these strains have been sequenced using short-read sequencing technologies, and the sequence reads from each strain have been aligned to their respective reference genomes. For this study, we extracted sequence reads that did not align to their respective reference genomes from 1,790 strains of *C. briggsae* , 1,631 strains of *C. elegans* , and 690 strains of *C. tropicalis* ( *Caenorhabditis* Natural Diversity Resource (CaeNDR) release 20231201). To taxonomically classify these unaligned reads, we used the k-mer-based classifier tool, Kraken2 (Wood et al., 2019), which creates k-mers from sequence reads and reduces the redundancy of k-mers by creating minimizers ( *i.e.* , non-redundant k-mers). Kraken2 uses minimizer-based indexing to query a user-specified database and return a proportion of matched minimizers to the lowest common ancestor of the matching reference sequences.

To benchmark Kraken2’s taxon classification accuracy, we extracted the unaligned sequencing reads from nine strains of *C. elegans* infected with a well characterized horizontally transmitted pathogen, microsporidia (Wadi et al., 2023), and the unaligned sequence reads from the bleached, microsporidia-free progeny of the same nine strains ( Figure 1A ). We found that Kraken2 had the highest power and lowest false discovery rate when using a minimizer match proportion of 10% against the Kraken2-compatible NCBI database, core_nt (database version 9/4/2024) (retrieved from https://benlangmead.github.io/aws-indexes/k2). Many strains from each species of *Caenorhabditis* had a diverse range of detectable non- *Caenorhabditis* genera from unaligned DNA sequencing reads. To reduce our search space, we filtered out the genera of *Caenorhabditis* , *Mus* , *Danio* , *Homo* , and *Escherichia* because they likely represent laboratory contaminants. Additionally, we filtered out genera of *Sphingomonas* , *Acinetobacter* , *Microbacterium* , and *Pseudomonas* because these genera are ubiquitous environmental bacteria that are horizontally transmitted and were not informative for identifying vertically transmitted endosymbionts. After this filtering, Kraken2 classified genera in 321 strains of *C. briggsae* (17.9%), 115 strains of *C. elegans* (7.1%), and 105 strains of *C. tropicalis* (15.2%) at a 10% minimizer match threshold ( Figure 1A ). We did not see any bacterial contaminants in obvious candidate genera, such as *Rickettsia* , *Wolbacchia* , or *Elrichia* (bacteria known to be vertically transmitted), even when we lowered the minimizer match threshold to 4%.

Because we did not detect any obvious candidate taxa, we prioritized *Caenorhabditis* strains that captured a range of bacterial taxa in order to maximize the chance of detecting potential endosymbionts experimentally. We excluded strains containing bacteria known to form endospores because they are more likely to be external contaminants that survived bleaching rather than intracellular residents. Selection was made at the level of individual *Caenorhabditis* strains, and the bacterial taxa detected in the genome assemblies were treated as the putative microbes to be validated. In total, seven strains were selected: ECA1191 ( *C. elegans* with *Achromobacter* sp., 11.69% minimizer match), NIC1781 ( *C. elegans* with *Brevundimonas diminuta* sp., 10.48%), NIC2011 ( *C. elegans* with *Pseudochrobactrum* sp., 12.84%), QG2833 ( *C. elegans* with *Cupriavidus cauae* , 59.78%), NIC1204 ( *C. briggsae* with *Myroides* sp. Bacteria, 11.8%), ED3032 ( *C. briggsae* with *Kocuria rhizophila,* 14.9%), and ECA1297 ( *C. elegans* with *Streptomyces* sp., 10.26%). Each of these strains were probed for bacterial infection using 16S ribosomal RNA FISH, using a universal bacterial probe EUB338 (Poirier et al., 2024; Rivera et al., 2022). None of the tested samples showed any obvious bacterial infection in the germline or any somatic tissue ( Figure 1B ). As a positive control, we fed N2 *C. elegans* with the microbiome bacteria MYb71 ( *Ochrobactrum pecoris* ) and were able to visualize bacteria in the gut lumen ( Figure 1B ), demonstrating that our validation procedure works. Note that the green fluorescence observed within embryos is a documented autofluorescence artifact associated with FISH protocols in gravid *C. elegans* hermaphrodites (Rivera et al. 2025), and no observed signal is consistent with bacterial morphologies, such as bacteria-shaped puncta (bacilli, coccobacilli, or cocci), in the germline tissue and/or the embryos ( Figure 1B ) *.* Because potential vertically transmitted microbes were able to be detected with Kraken2 but not experimentally detected using FISH probes, these results might be indicative of incomplete bleaching of wild strains or contamination during sequencing preparation, causing detectable levels of microbes that are not present in the animal. Alternatively, the number of minimizers needed to reach the percent minimizer match threshold will be different among wild strains based on the number of unaligned reads, causing different abundance levels of detectable taxa, even for wild strains that have the same percent minimizer match threshold for a given taxon.

Our experimental results do not identify any vertically transmitted microbes in free-living self-fertilizing *Caenorhabditis* species. As a future direction, developing improved *in silico* heuristics to better quantify the abundance of candidate vertically transmitted microbes in wild strains might improve strain selection and sensitivity of *in vivo* detection.

## Methods

*Taxonomic classification of unaligned reads*

Short-read sequencing alignment files used in this study are available at the CaeNDR, release 20231201 (Crombie et al., 2024). Wild strain sequence reads that did not align to the reference genome of each self-fertilizing species were extracted using samtools (v.1.19.2) (Danecek et al., 2021). Taxonomic classification of unaligned reads for every wild strain was performed using Kraken2 (v.2.1.3) (Wood et al., 2019) and the *core_nt* NCBI Kraken2 database (database version 9/4/2024) (retrieved from https://benlangmead.github.io/aws-indexes/k2). For control analysis, wild *C. elegans* strains ECA1388, ECA2679, ECA2680, ECA2681, ECA2682, ECA2683, ECA2684, ECA2685, and ECA2686 were microsporidia-infected when isolated from nature. These strains were whole-genome sequenced and cryopreserved. Then, these strains were spot bleached and their uninfected progeny were whole-genome sequenced and frozen as strains ECA1389, ECA1211, ECA1214, ECA1228, ECA1269, ECA1281, ECA1282, ECA1287, and ECA2803. Data visualization was performed using RStudio (v.4.2.1). All command-line scripts used for analysis and the R script used for visualization can be found at https://github.com/AndersenLab/vertTrans_microbe_discovery.

*Strain selection and maintenance*

Eight nematode strains ( *C. elegans* strains, ECA1191, NIC1781, NIC2011, QG2833, NIC1204, and ECA1297, and two *C. briggsae* strains, NIC1204 and ED3032) were selected so that the contaminating microbes represent a broad range of bacterial taxa, as described above. These *Caenorhabditis* strains were maintained on standard Nematode Growth Media (NGM) plates seeded with *Escherichia coli* OP50 incubated at 20°C. Once animals reached the gravid stage, they were spot bleached. L1s were plated on standard NGM plates seeded with OP50 for three days before conducting FISH.

*Alignment of 16S*

The DNA sequence for the small ribosomal RNA subunit (16S) for representative species for each taxa identified using Kraken2 were aligned using MUSCLE v.3.81551 with default parameters. This alignment was used to verify compatibility of the EUB338 probe (sequence: GCTGCCTCCCGTAGGAGT, custom synthesis obtained from Biosearch Technologies) used to detect bacterial infections.

*RNA Fluorescent in situ hybridization*

RNA FISH was performed as described previously (Rivera et al., 2025). For the EUB338 RNA FISH probe, hybridization was conducted overnight at 46°C and three washes were conducted at 48°C for 30 minutes each. Animals were mounted in Vectashield, antifade mounting media, with DAPI. The EUB338 FISH probe targeting the 16S rRNA of bacteria was conjugated to 5-Carboxyfluorescein (FAM). 

*Statistical analysis*

The statistical power of Kraken2 to identify microsporidia in the unaligned reads of microsporidia-infected strains was calculated as the number of times the genus *Nematocida* was identified in infected strains plus the number of non- *Nematocida* genera identified in non-infected strains, divided by the total number of genera identified (inclusive of NA - strains whose reads did not yield any classification were counted once). The false discovery rate (FDR) of Kraken2 was calculated as the number of non- *Nematocida* genera identified in an infected strain plus the number of *Nematocida* genera identified in non-infected strains, divided by the total number of genera identified (inclusive of NA - strains whose reads did not yield any classification were counted once). We chose a range of percent minimizer match cutoffs to optimize power and FDR, the data are reported as follows: percent minimizer match threshold = power:FDR (1 = 36.4:63.51, 3 = 44.4:55.5, 5 = 56:44, 10 = 73.68:26.32, 12 = 66.67:33.3, 15 = 58.8:41.2).

## Reagents

All strains used in experimentation are available upon request from the *Caenorhabditis* Natural Diversity Resource and probes used are described above.
